# Does having a similar disability matter for match outcomes?: A randomized study of matching mentors and mentees by visual impairment

**DOI:** 10.1002/jcop.22116

**Published:** 2018-07-27

**Authors:** Eline C. M. Heppe, Janis B. Kupersmidt, Sabina Kef, Carlo Schuengel

**Affiliations:** ^1^ Vrije Universiteit Amsterdam; ^2^ Innovation Research & Training

## Abstract

Mentoring is modestly effective for youth with a chronic illness or physical disability; however, program effectiveness may be enhanced when mentors and mentees are matched on shared interests and experiences. To test this hypothesis, mentees were randomly assigned to having a mentor with or without visual impairment (VI). Results showed that mentors without VI were younger and more likely to work or be educated in a helping profession and less likely to have a fixed mindset and extremely high positive expectations than mentors with VI. The main analyses on match outcomes showed that mentors with VI had significantly fewer and shorter match meetings, had a weaker relationship with their mentees, and were more likely to end their match prematurely. Mentor age, helping profession background, and fixed mindset were confounds in several analyses and reduced the significance of the relationship between VI group and match meeting quantity. The only relationship that remained significant controlling for covariates showed that matches including a mentor with VI were significantly more likely to end in premature closure than matches including a mentor without VI. Implications of the findings for future research and program practices related to matching were discussed.

## 1 INTRODUCTION

Social participation is challenging for people with visual impairments (VI). Hopes of meaningful relationships, engagement in leisure activities, and successful work and school careers are often thwarted by the difficulties associated with having VI. Young people with VI have relatively small social networks, have low social competence, spend more time at home, date less frequently, are less likely to start a family, and are more likely to have children at a later age than peers without VI (Gold, Shaw, & Wolffe, 2010; Kef, 1997; Kef & Bos, 2006; Kef, Hox, & Habekothe, 2000; McDonnall, 2010). Given the compromised social development of youth with VI, effective interventions are needed to enhance their social functioning and social participation.

### 1.1 Relationship‐Based interventions may be helpful

Relationship‐based interventions have the potential for helping youth with VI address and overcome their social developmental challenges. Vygotsky's (1986) theory explains how relationships are important to assist young people in advancing beyond their zone of proximal development such that relationships provide the mechanism of change for developmental progress. In addition, self‐determination theory posits that interpersonal relatedness provides a motivational context for effort, persistence, and growth (Deci & Ryan, 2000). Furthermore, Rhodes (2002) suggested that participating in a high‐quality (i.e., trusting, mutual, and empathic) mentoring relationship may be an effective means of supporting mentees’ social and emotional growth, such that mentoring can fill the gap between professional help and positive peer relationships. Thus, mentoring support can be helpful when mentees have limited experience in social activities and peer engagement and are at high risk for multiple negative developmental problems, as is commonly found in young people with disabilities such as VI.

In fact, the positive effects of mentoring have been demonstrated across a wide range of outcomes in youth (DuBois, Portillo, Rhodes, Silverthorn, & Valentine, 2011). Studies examining the effectiveness of mentoring for youth with a disability or chronic illness are rare, with even fewer studies testing the effect of mentoring on youth with VI. Studies suggest that mentoring youth with disabilities promotes independence and communication skills (Britner, Balcazar, Blechman, Blinn‐Pike, & Larose, 2006), improves self‐esteem and self‐advocacy skills (Maslow & Chung, 2013), enhances personal empowerment and coping skills (Shpigelman, Weiss, & Reiter, 2009), and increases academic and job successes and skills related to having a disability (McDonald, Balcazar, & Keys, 2005). Other positive outcomes from being mentored for youth with VI include increased decision‐making efficacy, positive personal hope for the future, and positive attitudes about their impairment (Bell, 2012). In addition, job‐seeking self‐efficacy, career adaptability, and assertiveness in job hunting are increased for mentored youth with VI (O'Mally & Antonelli, 2016). These studies are important, as they expand our knowledge about the effect of mentoring on youth at risk.

### 1.2 Matching

A meta‐analysis on the efficacy of mentoring programs examined whether variations in program practices were associated with larger effect sizes on youth outcomes (DuBois et al., 2011). When mentoring programs matched mentors and mentees on interest similarity, the effect size doubled from small (.20) to medium (.41) on youth outcomes compared with mentoring programs that matched on other criteria. Despite the effect of matching practices on program efficacy, few studies have directly examined the effect of match similarity on match or mentee outcomes.

Match similarity is itself a construct in need of conceptual definition. Eby and colleagues (2013) divided similarity into one of three categories, namely, *deep‐level* (similarity in attitudes, beliefs, values, or personality), *surface‐level* (similarity in race or gender), or *experiential* (similarity in experience‐based factors such as educational level, academic discipline, functional area, departmental affiliation, employment setting, job tenure, organizational setting, and geographic location). Deep‐level similarity had the strongest positive effect on mentees’ perceptions of instrumental and psychosocial support and relationship quality, followed by the effects of experiential similarity. Similarity with respect to surface‐level characteristics resulted in little to no effect on match outcomes. Eby and colleagues (2013) noted two methodological limitations in the meta‐analysis. First, none of the studies used an experimental design, limiting the ability to draw causal inferences, and second, most of the studies were about workplace mentoring, limiting the ability to generalize definitions of the three types of similarity to the youth mentoring literature.

The current study builds upon this literature on matching by utilizing an experimental design to examine the effects of matching on the basis of similarity on relationships involving youth with VI. In addition to matching mentors and mentees on the basis of similar hobbies and interests, it was hypothesized that matching on having a similar disability could further strengthen the positive effect of mentoring on match outcomes. Sharing a disability could result in mentors and mentees having similar kinds of life experiences across a range of domains (e.g., social, academic, vocational, leisure). Furthermore, these similar life experiences may lead to other kinds of similarities between mentors and mentees in their worldviews, beliefs, attitudes, and values. These similarities in experiences and cognitions could contribute to matches including both mentors and mentees who have VI having not only experiential similarities but also deep‐level similarities. Because both experiential and deep‐level similarities have been found to enhance match outcomes (Eby et al., 2013), matching based upon disability similarity was expected to enhance match closeness and effect.

One study examined the effect of matching on disability by randomly assigning students with one of several types of disabilities to have a mentor either with or without a disability or to a no‐treatment control group (Sowers et al., 2016). Although positive differences were found for youth in both of the mentoring conditions compared with those in the nonmentoring control group, matching on disability did not significantly affect outcomes. It is important to note that the participants had a wide range of disabilities, and mentors and mentees were matched based upon having similar disability challenges and mentee's preferences, but not on having the same disability. The null findings may have resulted from the fact that sharing the general experience of having a disability may have diluted the potential effect of having the same disability. Among mentoring interventions for people with VI, two studies matched mentees with similarly impaired mentors and found an increase in several career‐related outcomes and positive attitudes about impairment: However, neither study compared matches including mentors with VI to matches including mentors without VI (Bell, 2012; O'Mally & Antonelli, 2016).

Similarity with respect to disability status may help mentors with VI by being better prepared to take on the role of an effective mentor than mentors without VI. Because mentors can positively affect their mentee's development through the roles they play in their mentee's lives, such as being a positive role model, nurturer of possibilities, and trusted adult friend (Kupersmidt, Stelter, Rhodes, & Stump, 2017), sharing a disability may make it easier to contribute to at least two of these roles. An older peer or adult mentor with VI, who is socially resilient, can be a positive role model for a mentee with VI by sharing personal opinions about the importance and value of social support and demonstrating social and emotional competence. Mentors with VI can also leverage their history, social capital, life experiences, and adept social skills in their mentoring relationships with mentees with VI to nurture and cultivate their mentees’ paucity of social relationships and skills. Furthermore, sharing the disability of VI may diminish the risk of a mentor playing an inappropriate mentoring role due to having a better understanding of the risks, rewards, and practical challenges associated with VI.

In contrast to these helpful roles that define an effective mentoring relationship, engaging in inappropriate mentoring roles such as being a teacher, parent, therapist, peer friend, or adult advisor can negatively affect a mentee and a mentoring relationship (DuBois et al., 2011; Rhodes, Liang, & Spencer, 2009). Sighted mentors may have less of an understanding of the needs of mentees with VI and may play inappropriate roles with mentees with VI in an effort to be helpful. For example, individuals with VI have many mobility issues that can affect their participation in social activities outside of their homes (Berger, 2012; Kef et al., 2000). Sighted mentors may not understand the importance of mentees with VI learning how to navigate and travel alone outside of their homes to promote independence and autonomy and may provide too much assistance, thereby overstepping their mentoring role. Mentors with VI may have a better understanding of the boundaries or roles in their mentoring relationship compared with sighted mentors, which could positively affect match outcomes for mentoring relationships with mentors with VI.

### 1.3 Match outcomes

Match outcomes including the strength of a mentoring relationship and relationship quality are important predictors of youth outcomes. Specifically, mentees in high‐quality matches have better outcomes (Erdem, DuBois, Larose, Wit, & Lipman, 2016; Rhodes, Schwartz, Willis, & Wu, 2014), including better relationships with parents and teachers (Chan et al., 2013), and peers (Karcher, 2008), than mentees in lower quality relationships. In addition to the effect of the strength of the mentoring relationship on youth outcomes, match duration also is an important factor for determining match effect. Match length has been associated with youth outcomes (DuBois & Rhodes, 2006; Grossman & Rhodes, 2002) such that when match length increases, positive youth outcomes also increase. In fact, mentoring relationships that last for at least one year or longer show the greatest benefits; in contrast, matches that terminate before 6 months do not show clear benefits (Grossman, Chan, Schwartz, & Rhodes, 2012).

Despite the benefits of match longevity, premature match closure has been found in 30% to 50% of all formal mentoring relationships (DeWit et al., 2016; Kupersmidt, Stump, Stelter, & Rhodes, 2017) and premature closure is related to negative youth outcomes (Grossman & Rhodes, 2002; Herrera, Grossman, Kauh, Feldman, & McMaken, 2007; Spencer, 2007). Mentees who experience premature match closure can experience feelings of disappointment, guilt, anger, shame, and abandonment (Karcher, 2005). Because of the deleterious effects of premature match closure on youth outcomes, research has been conducted to study factors that may predict or cause premature closure in mentoring relationships.

Mentor characteristics such as age, work experience in a helping profession, marital status, and level of income have been shown to predict premature closure (DuBois, Holloway, Valentine, & Cooper, 2002; Grossman & Rhodes, 2002; Kupersmidt, Stump, et al., 2017). In addition, mentors with extremely high positive expectations can experience feelings of discouragement and frustration when they perceive a lack of improvement in their mentees or their mentoring relationships, which may result in their ending the mentoring relationship abruptly or prematurely (Madia & Lutz, 2004; Spencer, 2007). Similarly, match longevity may be affected when mentors’ motivations or goals are atypical or overly focused on addressing personal needs. For example, self‐enhancement as a motive for becoming a mentor was negatively related to relationship quality (Karcher, Nakkula, & Harris, 2005).

Based on the literature on volunteering, mentors with more altruistic motivations and goals may be more persistent and committed to the mentoring relationship than those with more self‐oriented motives (Clary & Orenstein, 1991; MacNeela, 2008). In addition, professionals serving in a role similar to that of a mentor who hold more of an entity theory provided less effective support to their mentees or clients (Meppelder, Hodes, Kef, & Schuengel, 2014; Rattan, Good, & Dweck, 2012). Thus, mentors’ implicit theories or mindset about the malleability of a person may affect match outcomes because positive attitudes toward personal growth can foster resilience, empathy, and perseverance in the context of a mentoring relationship.

### 1.4 Current study

The main purpose of this study conducted within the context of a larger project (Heppe, Kef, & Schuengel, 2015) was to examine the efficacy of matching mentees with VI with sighted mentors or with mentors who have VI. Using a rigorous experimental design, we tested the potentially important and generally understudied effect of matching practices on match outcomes. The main hypothesis was that sharing a disability, in this case VI, would be associated with better match outcomes.

## 2 METHOD

### 2.1 Participants

*Mentors*. A total of 50 mentors with and without VI were recruited to participate in this community‐based mentoring study. Three of the recruited mentors were not matched with a mentee because they did not live within geographic proximity to the homes of the mentees. Of the remaining 47 mentors, 11 were excluded from the study because their match with a mentee was not based upon randomization, resulting in 36 mentors participating in this study. Exactly 50% (N = 18) of the total group of mentors had VI (low vision or blind). During the study, five mentors were matched to more than one mentee after their first mentoring relationship ended prematurely. These five mentors were asked to report on only one of their mentoring relationships, specifically, the mentee with whom they had the longest mentoring relationship.

*Mentees*. Adolescents with VI were randomly assigned to one of three conditions, namely, having a mentor with VI, having a mentor without VI, or a care‐as‐usual control group (Heppe et al., 2015). The adolescents in the control group were not relevant to the current hypothesis. In total, 51 mentees were randomly assigned to the two experimental groups. Three mentees were not matched with a mentor because none of the mentors lived within geographic proximity to their homes. Of the remaining 48 mentees, nine were excluded from the analyses because the match was not based upon randomization; three were excluded from the analyses because their mentors did not report on their relationships. The mean age of the remaining N = 36 mentees participated in this study was 20 years (ranging from 17 to 23 years). Exactly 50% of them were female and 83% were born in the Netherlands. Almost 30% of the mentees were blind and 70% low sighted.

### 2.2 Measures

#### 2.2.1 Mentor report

*Mentor demographic and background characteristics*. We asked mentors about their age, sex, highest level of education, visual impairment status, involvement in a romantic relationship, educational or work experience in a helping profession, and employment status during a face‐to‐face or telephone intake interview. We asked about date of birth during the postintervention interview and used it to calculate each mentor's age.

*Mindset*. We administered an eight‐item questionnaire to assess implicit theories of the static (fixed) or malleable (growth) nature of a person. This questionnaire was a modified version (Meppelder et al., 2014) of the Mindset Questionnaire (Levy, Stoessner, & Dweck, 1998), adapted for assessing mindset regarding personal growth rather than solely focusing on mindset regarding malleability of intelligence. The measure comprises four items that assess a fixed mindset (e.g., “the kind of person someone is, is something basic about them, and this can't be changed very much”) and four items that assess a growth mindset (e.g., “everyone, no matter who they are, can significantly change their basic characteristics”). Participants rated the items on a 6‐point Likert scale ranging from 1 (*strongly disagree*) to 6 (*strongly agree*). Then we calculated a mean score for fixed mindset, with higher scores representing more of a fixed mindset. The internal consistency of the mean total fixed mindset items was .85.

*Expectations*. We used a translated version of the 12‐item Mentoring Expectations Questionnaire (Kupersmidt, Stelter, et al., 2017; modified from Madia & Lutz, 2004) to assess the prematch expectations of the mentors. The measure comprises two subscales: extremely high negative expectations (e.g., “I think my mentee will never respect my opinions”) and extremely high positive expectations (e.g., “I think my mentee will want my help right away”). Participants rated the answers on a 6‐point Likert scale ranging from 1 (*strongly disagree*) to 6 (*strongly agree*). In this study, we used 10 of the 12 original items. The internal consistency of the high negative expectations subscale was .69 and high positive expectations subscale was .77.

*Motives*. We used a translated version of the 23‐item Mentor Motivations Questionnaire (Kupersmidt, Stelter, et al., 2014; adapted from Allen, Poteet, & Burroughs, 1998, and Clary et al., 1998) to assess mentors’ prematch motives. The measure comprised four subscales: Over Involvement with Children (e.g., “it's easier for me to have relationships with children than with adults”); Civic Responsibility (e.g., “I want to give back to society”); Self‐enhancement (e.g., “It will make other people respect me”); and Social Reciprocation (e.g., “I want to be a friend to a child in the same way other adults were for me”). Participants answered questions on a 3‐point Likert scale (1 = no, this is not a reason why I decided to be a mentor, 2 = Yes, this is somewhat a reason why I decided to be a mentor, and 3 = Yes, this is definitely a reason why I decided to be a mentor). In this study, we used five out of the six original items to assess the Over Involvement in Children subscale (α = .65), 9 out 10 original items to assess Civic Responsibility (α = .65), and all three of the original items to measure Social Reciprocation (α = .75). The reliability of the subscale of Self‐enhancement was poor (α = .39); hence, it was not included in the analyses in this study.

*Length of match in days*. We calculated length in days based on the date of the first and the last meeting of every match. Mentors provided the meeting dates.

*Meetings*. We calculated the total amount of the meetings based on the total amount of evaluation forms completed, by each mentor after every meeting with his/her mentee.

*Length of meetings in hours*. We calculated the sum total length of time spent in match meetings by calculating the sum total number of hours reported spent in every match meeting. We gathered these data from the evaluation forms that the mentors completed after every meeting. When data were missing, we calculated the duration in hours based upon the mean number of hours spent during the other match meetings.

*Strength of the relationship*. Participants completed a translated version of the 14‐item Mentor Strength of Relationship Scale (e.g., “I am enjoying the experience of being a mentor”; Rhodes et al., 2014). This scale was designed to measure the strength of the mentoring relationship as reported by mentors. Participants rated the answers on 5‐point Likert scale ranging from 1 (*strongly disagree*) to 5 (*strongly agree*). The internal consistency of the scale was good (α = .81).

*Perceived value of match similarity with respect to VI*. The research assistants conducted the postmatch structured telephone interviews, during which mentors were asked if they thought that sharing the same disability added value to being a mentor of a mentee with VI (yes or no) and, if so, why. The first author coded the responses to the open‐ended question based upon each unique theme mentioned in the answers. Once a comprehensive set of coding categories was created, the first author coded all additional responses into these categories. The first author explained the coding categories and examples of codes to the second author. The responses were translated into English and the second author coded the English versions; rater agreement was 82%. We noted the most frequently used themes to provide subjective impressions of the relevance and value of shared disability on match closeness.

#### 2.2.2 Mentee report

*Strength of the relationship*. We used a translated version of the 10‐item Youth Strength of Relationship Scale (Rhodes et al., 2014; e.g., “When I am with my mentor, I feel safe”) to assess mentee's perceptions of the strength of their mentoring relationships. Participants rated the answers on a 5‐point Likert scale ranging from 1 (*strongly disagree*) to 5 (*strongly agree*). The internal consistency of the scale was.95.

### 2.3 Procedure

*Mentors*. A national recruitment campaign has been conducted throughout the Netherlands with support from a wide variety of organizations. City‐based volunteer recruitment agencies, rehabilitation centers, community sports leagues designed for individuals with VI, and universities disseminated information about the study through word of mouth and social media channels. Mentors learned about the study on a project recruitment website where they could enroll to participate by completing a brief online eligibility application. They indicated their age, presence or absence of VI, home address, and occupation.

In this study, eligibility criteria for being accepted as a mentor included age (18–45 years) and either having VI or not having any type of disability. We invited all initially eligible volunteers to be interviewed in a second round of screening. The structured eligibility interview contained questions about levels of social participation at work, at leisure, with peers, with family members, and in romantic relationships; life experiences; understanding of the potential value of mentoring to mentors and mentees; expectations for being a mentor, future mentees, and the mentoring relationship; and level of commitment to the minimum frequency and length required by the mentoring program.

After the screening interview, we conducted a team meeting with the first and third authors and other research staff to discuss each candidate. Exclusionary criteria included pervasive lack of social participation, seeking a mentee to compensate for loneliness or to meet personal needs, self‐reported trauma exposure, having mental health problems, and being overcommitted. The research staff shared input on their observations with the investigators, who consensually decided upon who to include and who to exclude from the study.

We emailed eligible volunteers information about the goals and requirements of the study and asked them to sign an informed consent form. Potential mentors who consented to participate in the study were invited to attend two, 4‐hour, in‐person, instructor‐led training sessions that were conducted a week apart. Admission into the study was rolling culminating in two cohorts of mentors being trained.

During the prematch training workshop, volunteers completed prematch questionnaires, and questions were read out loud to the whole group. Trained research assistants helped mentors with VI to complete the questionnaires and wrote down the mentors’ responses. Consistent with the Screening Standard in the *Elements of Effective Practice for Mentoring* (EEPM; Garringer, Kupersmidt, Rhodes, Stelter, & Tai, 2015), the research team used the prematch training sessions to continue to observe and interact with potential mentors to screen them for their appropriateness for participating in the intervention. Research assistants observed the behavior of potential mentors during the training and kept comprehensive notes about their observations that they submitted to the investigators. They were asked to observe any unusual, antisocial, or especially withdrawn behaviors of the potential mentors.

Based upon these observations and the experiences of the investigators of volunteers during the training, four volunteers were excluded from the study. After the training workshop, mentors submitted a certificate of good conduct (i.e., requested by their own city office, which declares that they had not committed any criminal offences relevant to performing their mentoring activities) and were matched with a mentee. We conducted postintervention interviews over the phone as soon as possible after the match closed, which were scheduled at the convenience of the mentor. The mean number of days between closure and the postintervention interview was 110 days and ranged from 1 day to 536 days. Although we did not provide incentives for completing questionnaires, we provided mentors with a financial budget of 420 Euros to support the costs of their match activities with their mentees. In addition, mentors had an unlimited local transportation budget for taking their mentees on outings. Mentors submitted receipts for reimbursement of all travel and activity expenses.

*Mentees*. We recruited adolescents with VI through two national service organizations, as well as through online banners, brochures on social media, and Internet advertisements. By signing an informed consent, participants agreed to be allocated to one of the two experimental groups or the care‐as‐usual control group. We used randomization stratification on geographical proximity with a block size of 15 participants to randomly assigned participants. Then we matched participants assigned to one of the two experimental groups (mentees) to a mentor. Detailed open access descriptions of the rigorous preregistered experimental design are described in detail in Heppe et al. (2015).

*Matching*. After randomization, we first performed matching based upon the geographic proximity of the homes of mentors and mentees to one another and then upon having similar interests. Matches were homogeneous with respect to race and ethnicity. Both same‐ and cross‐gender matches were created. We informed matches that the maximum number of match meetings is 12 at a desired frequency of monthly meetings, and that they could extend their relationships to last a maximum of 18 months, if unforeseen events happen that make it impossible to have monthly meetings. The Ethics Committee of the Vrije Universiteit Amsterdam approved this study protocol, and reporting conformed to the CONSORT statement (CONSORT 2010 statement; Schulz, Altman, & Moher, 2010). Matching procedures are described in detail in Heppe et al. (2015).

*Intervention*. The mentoring intervention program was designed for the purpose of this study (Heppe et al., 2015) and involved one‐to‐one, community‐based mentoring comprising 12 meetings occurring over a period of 12 months. Mentors and mentees jointly planned their meeting dates, times, and locations to be held in locations that were convenient for the mentees and near the homes of the mentees. Match meetings could be scheduled to last for a maximum of one day or 8–12 hours. Activities performed during the meetings were based on the mentees’ goals and interests. Matches lacking inspiration to come up with activities could consult the Mentor Support Handbook for ideas. The handbook contained examples of activities, assignments, and exercises: visiting a movie theater, library, or art gallery; inviting friends for a high‐tea or preparing a meal for friends; participating in sports; and discussing goals over lunch, dinner, or drinks. We informed the mentors that the goal of their mentoring relationship was to promote their mentees’ participation in leisure activities, social relationships, and school or work.

## 3 RESULTS

### 3.1 Overview of analyses

First, we compared the two mentor groups on their demographic and background characteristics. Second, we compared the mentor groups on their prematch cognitions including their mindset, mentoring expectations, and mentoring motivations. Third, when analyses revealed group differences in a baseline mentor characteristic, we examined the baseline characteristic in individual analyses as a predictor of each match outcome. Fourth, we conducted univariate analysis of variance and logic regression analyses for the main analyses to test for differences between the two mentor groups on match outcomes. We examined baseline mentor characteristics that were significantly related to a match outcome in subsequent analyses as possible confounders of the relationship between VI Group and the match outcome.

### 3.2 Pretest descriptive statistics

As shown in Table 1, differences were found between mentors with and without VI on two variables. Mentors with VI were significantly older than sighted mentors. Age was negatively related to total number of match meetings, *F*(1,34) = 5.98, *p* < .05, such that older mentors were more likely to have fewer match meetings than younger mentors. In addition, mentors with VI were significantly less likely than sighted mentors to have been employed or educated in a helping profession. Having a helping profession background was related to a larger number of match meetings, *F*(1,34) = 4.49, *p* < .05, mentors’ reports of closer mentoring relationships, *F*(1,32) = 5.37, *p* < .05, and less likelihood of premature closure, *Χ* ^2^(1) = 5.84, *p* < .05, than not having a helping profession background.

**Table 1:** Demographic and background characteristic differences between mentor groups (N = 36)

	Mentors with VI N = 18		Mentors without VI N = 18				
Mentor characteristics	*M*	*SD*	*M*	*SD*	*F*(1)	*p*	*d*
Age (years)	33	5.54	28	5.28	7.68	.009	.94

	Mentors with VI		Mentors without VI				
	%	N	%	N	X^2^(1)	*p*	Phi
Male	44%	8	28%	5	1.08	.30	.17
Romantic relationship	56%	10	67%	12	0.47	.49	.11
Bachelor's degree or higher	61%	11	78%	14	1.18	.28	.18
Full‐time employment	28%	5	28%	5	0.00	1.00	.00
Part‐time employment	39%	7	67%	12	2.77	.10	.28
Employment or educated in a helping profession	17%	3	61%	11	7.48	.006	.46

*Note*. *M* = mean; *SD* = standard deviation.

### 3.3 Prematch cognitions

As shown in Table 2, mentors with VI differed from sighted mentors on prematch cognitions. Before matching, mentors with VI had more of a fixed mindset than mentors without VI had. Having more of a fixed mindset was associated with fewer match meetings, *F*(1,34) = 6.10, *p* < .05, briefer match meetings, *F*(1,33) = 8.21, *p* < .05, and greater likelihood of premature closure, *X^2^*(1) = 5.62, *p* < .05, than having less of a fixed mindset. Mentors with VI had more extremely high positive expectations then sighted mentors. The variable of having extremely high positive expectations was not significantly related to any of the match outcome variables. In addition, the two groups of mentors did not differ on the prematch cognitions of having extremely high negative expectations or on having motivations that were based upon overinvolvement with youth, civic responsibility, or social reciprocation.

**Table 2:** Prematch cognitions of mentors (N = 36)

	Mentors with VI N = 18		Mentors without VI N = 18				
Mentor cognitions	*M*	*SD*	*M*	*SD*	*F*(1)	*p*	*d*
Fixed mindset	3.53	0.52	3.03	0.68	6.11	.02	.84
Expectations: Unrealistically positive	3.21	0.45	2.74	0.55	7.67	.009	.94
Expectations: Unrealistically negative	1.85	0.50	1.89	0.49	0.65	.80	.09
Motivation: Overinvolvement with youth	1.53	0.41	1.54	0.51	.008	.92	.00
Motivation: Civic responsibility	2.25	0.23	2.40	0.20	3.90	.06	.67
Motivation: Social reciprocation	2.06	0.64	1.89	0.58	0.67	.42	.29

*Note*. *M* = mean; *SD* = standard deviation.

### 3.4 Match outcomes

Table 3 shows the means and standard deviations or percentages for the two mentor groups on each match outcome. Table 4 shows the results of the main analyses examining the effect of VI Group and potential confounding variables on each match outcome. Analyses revealed no difference between the two groups on the length of their mentoring relationships in days. Without covariates, mentors with VI had significantly fewer match meetings, shorter match meetings, and reported a weaker relationship with their mentees than sighted mentors. None of the covariates (i.e., age, helping profession, fixed mindset) was a significant predictor of match meeting quantity when the VI group was in the model; however, the VI group was also no longer significantly related to match meeting quantity when the covariates were added to the model.

**Table 3:** Descriptive statistics of match outcomes (N = 36)

	Mentors with VI		Mentors without VI	
	N = 18		N = 18	
Match outcomes	*M*	*SD*	*M*	*SD*
Relationship length (days)	260	157.45	362	188.84
Total number of match meetings	5	3.20	9	4.67
Length of match meetings (hours)	19	18.58	37	24.22
Strength of relationship: Mentor report	3.12	0.61	3.64	0.58
Strength of the relationship: Mentee report	4.24	0.73	4.04	0.71

	Mentors with VI		Mentors without VI	
	%	N	%	N
Premature closure	89%	16	44%	8

*Note*. *M* = mean; *SD* = standard deviation.

**Table 4:** Match outcomes as a function of visual impairment of mentors group with and without covariates (N = 36)

			Test of predictors			Model Test		
Match outcomes			*F*(1)	*p*	*d*	*F*(2)	*p*	*d*
Relationship length (days)	Model 1	VI	3.05	.09	0.61			
Total number of match meetings	Model 1	VI	5.64	.03	0.81			
	Model 2	VI	2.26	.15	0.52			
		Age	2.57	.12	0.56	4.23	.03	1.01
	Model 3	VI	2.49	.13	0.55			
		Helping profession	1.46	.24	0.42	3.59	.04	0.93
	Model 4	VI	2.51	.13	0.55			
		Fixed mindset	2.88	.10	0.59	4.41	.02	1.03
Length of match meetings (hours)	Model 1	VI	6.06	.02	0.87			
	Model 2	VI	2.41	.13	0.55			
		Fixed mindset	4.13	.05	0.74	5.49	.01	1.19
Strength of relationship: Mentor report	Model 1	VI	6.34	.02	0.91			
	Model 2	VI	2.63	.12	0.58			
		Helping profession	1.78	.20	0.48	4.14	.03	1.03
Strength of the relationship: Mentee report	Model 1	VI	0.56	.46	0.28			

			Beta	*p*	OR	*Χ* ^2^(1)	*p*	
Premature closure	Model 1	VI	−2.30	.01	.10			
	Model 2	VI	−1.88	.05	.15			
		Helping profession	−1.15	.18	.32	10.34	.01	
	Model 3	VI	−1.93	.04	.15			
		Fixed mindset	−0.97	.19	.38	10.56	.01	

Mentors with VI had match meetings that were shorter in duration than sighted mentors had. The VI group was no longer significant when fixed mindset was added to the model. Notably, fixed mindset was significantly related to match meeting duration with the VI group in the model. Mentors with VI reported a weaker, less close relationship with their mentees than sighted mentors. When the helping profession background variable was added to the model, both VI group and helping profession background were no longer significantly related to mentor report of relationship strength. There was no difference between VI groups on the strength of the mentoring relationship according to mentees. Matches including mentors with VI were significantly more likely to end in an unanticipated closure compared with matches including sighted mentors. This relationship between the VI group and premature match closure was significant even when the potential confounders of helping profession background and fixed mindset were added to the models.

### 3.5 Perceived value of a shared disability

Of the total group of 36 mentors, two sighted mentors did not complete the postintervention interview. Of the mentors, 71% (24 out of 34) reported that sharing the same disability added value to being a mentor. Notably, almost all of the mentors with VI (16 out of 18) reported that sharing the same disability added value to being a mentor of a mentee with VI; however, only half (8 out 16) of sighted mentors shared this opinion. The main reason why mentors thought that having a shared disability added value was that a mentor with VI could share their disability‐related experiences with their mentees, which sighted mentors could not do. As one mentor with VI stated, “When you have to overcome the same obstacles, it is more easy to share your thoughts, ideas, and thus, experiences.”

In addition, mentors stated that having a similar disability would make it easier to share knowledge about topics such as the latest technological devices or support programs for people with VI. For example, mentors and mentees with VI may use the same aids for public transportation; thus, they may share the same travel routines and experience similar types of accessibility challenges when completing career, educational, and leisure activities. Among those who were in favor of matches sharing the same disability, three mentors mentioned that there are some orientation and mobility strategies that can be shared when both members of the match have VI.

Mentors explained that mentors with the same disability also may be more empathic because of having shared similar personal experiences with mentees with VI. For example, one mentor said: “We know the feeling of being disappointed. We know the struggles. We had and still sometimes have exactly the same feelings. For me, seeing my mentee or listening to my mentee's stories is like de‐ja‐vu.” In addition, mentors stated that this kind of understanding could lead to increased trust, shared attitudes, and common interests.

About one third of mentors reported that they did not think that sharing the same disability would add value to being a mentor (10 out of 34). Specifically, 11% (2 out of 18) of mentors with VI and 50% (8 out of 16) of sighted mentors did not perceive that sharing a VI would add value. In addition, these mentors reported that the practical implications of matching people with VI could present a big recruitment challenge to mentoring programs. They also thought that the ability to be a good mentor was less about sharing disability‐related experiences and more about having “something” more general in common with one another. For example, one mentor stated: “I don't think that sharing the same disability would lead you to be a better mentor. It's more about just sharing one or two things in common. This could be the disability but also that you both like horses or that you both have been bullied during elementary school.”

## 4 DISCUSSION

This study was designed to test the effect of a key program practice, matching based upon similarity, on match outcomes using a rigorous experimental design. Previous research had suggested that matching based upon similarity was one of the main causes of match success; however, to the best of our knowledge, this hypothesis has not been experimentally tested with respect to similarity based upon disability status. Thus, this was the first study to randomly assign mentees to mentors with a similar disability or mentors without a similar disability. Although match similarity has been repeatedly found to be associated with both positive mentee and match outcomes (DuBois et al., 2011; Eby et al., 2013), the results from this study did not provide support for the hypothesized relationship between match similarity with respect to visual impairment (VI) and match outcomes. In fact, to some extent, the opposite pattern was found. Similarity with respect to VI did not enhance match outcome results and it significantly diminished some of the match outcomes. Mentees with VI who were randomly assigned to mentors who also had VI had matches that were more likely to end prematurely than matches including mentors who did not have VI.

Despite the lack of empirical support for the advantages of disability similarity, it is notable that almost two thirds of the mentors in this study believed that disability similarity would significantly and positively contribute to match and youth outcomes. Although the present study examined only match outcomes, the quantitative data suggested that disability similarity may be less important than commonly thought and did not confirm the hypotheses regarding the benefits of matching on the basis of disability similarity. Consistent with these findings on the lack of a positive effect of disability similarity on outcomes, a recent study examined youth outcomes for mentees with a disability and reported no differences between youth matched with a mentor having similar disability challenges and youth matched with a mentor who had no disability (Sowers et al., 2016). Taken together, these studies suggest that other factors or other similarity characteristics besides shared disability status may be more important for producing positive match and youth outcomes.

The large percentage of matches that closed prematurely was surprising given the fact that significant efforts were made to ensure that the mentors in this study, both those with and without VI, would be effective, prepared, safe, and suitable for serving as mentors to youth with VI. Volunteers were extensively interviewed, screened, and trained before being accepted into the study and matched with a mentee. Consistent with the Screening Standard in the EEPM (Garringer et al., 2015) and previous research on “deep‐level” similarity (Eby et al., 2013), matching within randomized conditions was predicated on shared vocational or leisure interests. All matches also received extensive, ongoing match support that was conducted at a minimum frequency of once a month, consistent with the Monitoring and Support Standard in the EEPM (Garringer et al., 2015). Suggestions to mentors and mentees during match support conversations were the result of consensual team discussions that included input from both practitioners and research experts. Unfortunately, these enhanced program practices (i.e., prematch training, matching on interest similarity, ongoing relationship support, and scaffolding) did not mitigate against the deficiencies found in matches involving mentees with VI.

This study further contributes to the growing literature on the role of similarity in promoting positive relationship development, suggesting that this process may be more complicated and nuanced than originally hypothesized. One explanation for the findings is that although experiential and deep‐level similarity has been consistently found to be positively related to mentoring outcomes (Eby et al., 2013), disability similarity may not represent experiential or deep‐level similarity, as originally thought. Disability similarity may operate as more of a superficial “social address” factor, rather than a form of experiential or deep‐level similarity, and may not strongly correspond to shared personalities, interests, or goals, as originally thought.

Other factors may supersede the effect of disability similarity on match outcomes for special populations of youth, such as those with VI. For example, matches that comprise mentors and mentees who both have a VI face added challenges that are associated with the VI itself. Specifically, having VI can result in restricted mobility (Berger, 2012) and has been found to be related to problems with social competence (Gold et al., 2010; Kef & Bos, 2006). If one or more of these practical or interpersonal challenges are present in both members of the match, then these challenges may mitigate against the potential positive effect associated with disability similarity and potential benefits of having had similar life experiences related to VI. A positive effect of similarity was not attenuated by disability similarity; in fact, the opposite was true in that negative match outcomes increased. More research should be conducted to understand better if disability may or may not lead to a shared experiential base.

Mentors with VI appeared to have some limitations in their ability to be effective community‐based mentors in one‐on‐one relationships with mentees with VI in terms of match length and strength. Some of the prematch differences between the two groups of mentors may help to explain these group differences. First, mentors with VI had higher scores on having a fixed mindset than mentors without VI. This difference between groups may reflect the fact that mentors with VI have a permanent physical disability and their life experiences associated with this impairment may limit their beliefs about the ability of their mentees to be able to change and grow. In addition, people with a more fixed mindset are less resilient (Dweck, Chiu, & Hong, 1995), so when faced with challenges in the early testing phase of their mentoring relationships, they may give up more easily (Spencer, Basualdo‐Delmonico, Walsh, & Drew, 2017). This may help to explain why they had fewer and briefer match meetings and shorter mentoring relationships.

Second, mentors with VI expected their mentoring relationships to be close and extremely positive more quickly compared with mentors without VI. These types of high positive expectations have been found to be related to higher rates of early relationship closure (Madia & Lutz, 2004; Spencer, 2007). Hence, when people having these high positive expectations hit inevitable “bumps in the road,” they may become disappointed and quit prematurely (Spencer et al., 2017). Some implications of these findings are that mentors with VI may need more intensive prematch training and support focused on better defining mentoring, building a growth mindset, and helping them to have expectations that are more realistic.

The longer and stronger relationships found in matches with sighted mentors reflect the fact that these mentors were more likely to have been employed or educated in a helping profession. These findings are consistent with previous findings on the possible advantages of being involved in a helping profession for being an effective mentor (DuBois et al., 2002). Previous exposure to the education or training received in a helping profession can help mentors with calibrating their expectations, coping with the challenges that can naturally evolve in the course of a helping relationship, and avoiding or managing boundary violations. These skills are especially important when mentoring a mentee with VI because stressors that are uniquely related to this particular disability (e.g., having other health‐related challenges, parental overprotectiveness, dependence, difficulty in mobility, lower social competence) may place constraints on the development of a strong mentoring relationship. These skills may help explain why individuals with a helping profession background had more match meetings, as well as longer and stronger mentoring relationships.

Although better match results for sighted mentors on total number of match meetings were found in independent analyses, age served as a confounding variable when added to the model. A possible explanation could be that with smaller age differences between mentors and mentees, it may be easier for the mentor to share power with his or her mentee, approach the relationship from a developmental as opposed to instrumental perspective, and avoid acting in a more hierarchical or parent‐like role. This finding is consistent with Levinson's (1978) recommendation that mentors should be a maximum of one‐half a generation older than their mentees. Although the positive effect of smaller age differences between mentors and mentees has been reported previously, better match outcomes for younger mentors have not been found for studies of general populations of mentees (Grossman & Rhodes, 2002; Grossman et al., 2012).

Surprisingly, no difference was found between mentees mentored by mentors with and without VI on their ratings of the strength of their mentoring relationships. Despite the lower reports on the strength of the mentoring relationship among mentors with VI, the smaller number of match meetings, and the shorter the durations of these meetings, mentees with VI felt their relationships were just as strong as relationships involving sighted mentors. This finding is not consistent with recent research that showed that a higher frequency of match meetings and longer lengths of match duration are associated with higher relationship quality (Eby et al., 2013; Herrera et al., 2007; Rhodes et al., 2014). Perhaps practical limitations, such as restrictions in mobility, orientation, and fatigue, limit the ability of mentors with VI to meet more frequently and arrange meetings of longer duration with their mentees. Nonetheless, these challenges did not compromise the ability of mentors with VI to establish an equally strong, close relationship with their mentees as mentors without VI, according to their mentees. Thus, although many of the indices of match outcomes were more negative for matches including mentors with VI, other indicators of mentoring success were observed in these matches. In addition, several of the findings for diminished effects of disability similarity disappeared when confounding variables were added to the models.

### 4.1 Limitations

Although the study's randomized design was a particular strength that addressed many threats to validity, the relatively small sample size may limit the generalizability and replicability of the findings. Furthermore, the fact that half of the sighted mentors were employed or educated in a helping profession and were significantly younger than the mentors with VI also may limit generalizability of findings. This hypothesis can be addressed in a replication in a larger randomized study that may be more balanced with respect to these confounding variables.

In addition, the low prevalence of VI in the general population made it impossible to match mentors and mentees on their exact VI. Although on the surface having VI is a specific disability, there is in fact much heterogeneity among people with VI. VI encompasses both blindness and low vision, and low vision can be severe or moderate. In addition, some people have been blind since birth, whereas others may have lost their vision gradually or later in life. Some people have blurred vision, and other may have darkness or blind spots in their field of vision. Matching based on these more specific characteristics of VI may be better for creating a match that has the possibility of either experiential or deep‐level similarity and forms a direction for future research.

### 4.2 Implications for research and practice

Deep‐level similarity has been associated with a wide range of match and youth outcomes. Therefore, an important direction for future research is to better define what similarity in values, beliefs, attitudes, and personality means and how to define these terms operationally. Additional studies with larger samples are needed to test the effect of matching based upon different types of similarity. These studies may help to illuminate the processes through which experiential and deep‐level similarity in a mentoring match can be assessed and used to improve program outcomes.

The surprisingly high rates of premature closure among matches with a similar disability could be further explored in future qualitative and quantitative research in order to gain insight into the process of relationship dissolution across a wide variety of matches. Some directions for future research are as follows: examining the source(s) (i.e., mentor, mentee, mutual, family member) and reasons (e.g., preventable reasons such as unmet expectations or goal) for closure; exploring the differences between successful and unsuccessful matches; investigating the challenges experienced by mentors and mentees with VI in establishing and maintaining a mentoring relationship; and studying mentoring program practices that may help to initiate and sustain mentoring relationships that include mentors or mentees with a disability (e.g., enhanced mentor and mentee training; increased frequency of coaching or match support meetings).

Mentees’ reports of relationship quality as a function of mentor group did not differ, suggesting that mentees felt satisfied and close to their mentors, regardless of whether or not their mentors shared having VI. Because this study examined only match outcomes, an important direction for future research is to obtain a more comprehensive picture of the efficacy of disability matching by examining its effect on a range of mentee and mentor outcomes.

From a practical perspective, this study demonstrated that enhanced prematch mentor training may be needed for mentors with VI matched with mentees with VI. Recent research showed that mentors who had been trained in mentoring roles and expectations were more knowledgeable, prepared, and self‐efficacious compared with training‐as‐usual mentors (Kupersmidt, Stelter, et al., 2017). Increased knowledge about these topics acquired in evidence‐based, prematch training programs may help mentors with VI to set boundaries more easily in their mentoring relationships, have expectations that are more realistic, and disclose risky or problematic situations to their mentoring program more quickly. Another reason that more specifically designed training for mentors with disabilities may be needed is that they were more likely to have a fixed mindset. Because person's mindset can be changed, mentors with a fixed mindset may need targeted prematch training and ongoing support to promote more of a growth mindset to support match longevity.

Mentors with experience working in a helping profession may have received training on topics such as relationship boundaries, realistic expectations, resolving conflict, and empathic listening. Thus, they may be better able to establish mentoring relationships that are closer and more enduring (DuBois et al., 2002). Future mentoring programs, especially those serving youth with a disability, could consider focusing their recruitment and screening efforts on locating and enrolling mentors with previous educational or vocational experience in a helping profession. In addition, mentor training and support may include topics, issues, and practices that parallel the methods used in helping professions, particularly for mentors who do not have education or previous experience in a helping profession.

A new approach to youth mentoring that should be examined in future research with youth with VI can capitalize on their existing social networks, and encourage them to cultivate mentoring or helping relationships with caring adults in their lives. In these types of informal mentoring relationships, instead of a mentoring program formally matching mentors and mentees to one another, mentoring would evolve more naturally and be more youth‐directed. These nonassigned relationships may still be formally enrolled in and monitored by a mentoring program, as in the National Guard Youth Challenge Program (Schwartz, Rhodes, Spencer, & Grossman, 2013), such that mentors and mentees are screened, trained, and supported. Some studies have shown natural and youth‐initiated mentoring relationships to be longer in duration, higher in quality, and less likely to end prematurely (Eby et al., 2013; Schwartz et al., 2013).

This strategy of training and supporting youth to identify potential mentors in their communities is a new direction for both research and practice in mentoring that may be particularly effective for community‐based and rehabilitation intervention programs serving vulnerable youth, such as youth with VI. Because youth‐initiated mentoring provides mentees with the possibility of selecting mentors from their own social networks who may already be familiar with the challenges associated with an impairment, this may be an optimal approach for youth with VI. In addition, this mentoring model may be more suitable for this population of youth, given the restricted mobility and transportation challenges that are often faced by youth with VI. Mentor training and support to complement this familiarity may lead to matches that are more effective and of higher quality.

### 4.3 Conclusion

Overall, this experimental study showed that matching mentors and mentees based on having a VI did not enhance match outcomes of length, duration, and strength (as reported by the mentor) of the relationship. In contrast, matching on disability status had a negative effect on match length being associated with high risk for premature closure, suggesting that the hypothesized positive effects of disability similarity on mentoring cannot be presumed. Prematch differences between groups including the fact that mentors with VI were more likely to have a fixed mindset and extremely high positive expectations for their relationships, older, and less likely to be educated or working in a helping profession than mentors without VI also contributed to match outcomes. Future research and practice on assigned mentoring relationships should continue to evaluate matching definitions and procedures as well as the effect of matching based upon disability similarity on youth outcomes, in order to better understand how to create matches with the greatest likelihood of producing relationship success.
